# IGFBP3 Colocalizes with and Regulates Hypocretin (Orexin)

**DOI:** 10.1371/journal.pone.0004254

**Published:** 2009-01-22

**Authors:** Makoto Honda, Krister S. Eriksson, Shengwen Zhang, Susumu Tanaka, Ling Lin, Ahmad Salehi, Per Egil Hesla, Jan Maehlen, Stephanie E. Gaus, Masashi Yanagisawa, Takeshi Sakurai, Shahrad Taheri, Kuniaki Tsuchiya, Yutaka Honda, Emmanuel Mignot

**Affiliations:** 1 Sleep Disorder Research Project, Tokyo Institute of Psychiatry, Setagaya, Tokyo, Japan; 2 Center for Narcolepsy, Stanford University, Palo Alto, California, United States of America; 3 Department of Neurology, Stanford University, Palo Alto, California, United States of America; 4 Department of Pathology, Oslo University, Oslo, Norway; 5 Molecular Genetics Department, University of Texas, Dallas, Texas, United States of America; 6 Department of Molecular Neuroscience and Integrative Physiology, Kanazawa University, Kanazawa, Japan; 7 Laboratories for Integrative Neurosciences and Endocrinology, Bristol University, Bristol, United Kingdom; 8 Tokyo Metropolitan Matsuzawa Hospital, Tokyo, Japan; 9 Japan Somnology Center, Neuropsychiatric Institute, Tokyo, Japan; 10 Howard Hughes Medical Institute, Stanford University, Palo Alto, California, United States of America; University of Cambridge, United Kingdom

## Abstract

**Background:**

The sleep disorder narcolepsy is caused by a vast reduction in neurons producing the hypocretin (orexin) neuropeptides. Based on the tight association with HLA, narcolepsy is believed to result from an autoimmune attack, but the cause of hypocretin cell loss is still unknown. We performed gene expression profiling in the hypothalamus to identify novel genes dysregulated in narcolepsy, as these may be the target of autoimmune attack or modulate hypocretin gene expression.

**Methodology/Principal Findings:**

We used microarrays to compare the transcriptome in the posterior hypothalamus of (1) narcoleptic versus control postmortem human brains and (2) transgenic mice lacking hypocretin neurons versus wild type mice. Hypocretin was the most downregulated gene in human narcolepsy brains. Among many additional candidates, only one, insulin-like growth factor binding protein 3 (IGFBP3), was downregulated in both human and mouse models and co-expressed in hypocretin neurons. Functional analysis indicated decreased hypocretin messenger RNA and peptide content, and increased sleep in transgenic mice overexpressing human IGFBP3, an effect possibly mediated through decreased hypocretin promotor activity in the presence of excessive IGFBP3. Although we found no IGFBP3 autoantibodies nor a genetic association with IGFBP3 polymorphisms in human narcolepsy, we found that an IGFBP3 polymorphism known to increase serum IGFBP3 levels was associated with lower CSF hypocretin-1 in normal individuals.

**Conclusions/Significance:**

Comparison of the transcriptome in narcolepsy and narcolepsy model mouse brains revealed a novel dysregulated gene which colocalized in hypocretin cells. Functional analysis indicated that the identified IGFBP3 is a new regulator of hypocretin cell physiology that may be involved not only in the pathophysiology of narcolepsy, but also in the regulation of sleep in normal individuals, most notably during adolescence. Further studies are required to address the hypothesis that excessive IGFBP3 expression may initiate hypocretin cell death and cause narcolepsy.

## Introduction

Narcolepsy-cataplexy is a common sleep disorder affecting 0.02–0.16% of the general population in the United States, Europe and Asia. Disease onset may be insidious or abrupt, typically occurring around adolescence, and is characterized by excessive daytime sleepiness, cataplexy (sudden loss of muscle tone triggered by emotions) and other manifestations of abnormal Rapid Eye Movement (REM) sleep.

Narcolepsy has characteristic biological markers including Human Leukocyte Antigen (HLA) association and dysfunction of hypocretin (also called orexin) neurotransmission. Almost all patients with narcolepsy-cataplexy share a common HLA allele, DQB1*0602 [1] suggesting an autoimmune basis for the disorder. Over 90% of narcolepsy-cataplexy cases are associated with a dramatic decrease in hypocretin-1 (HCRT1) in the cerebrospinal fluid [2].

Hypocretin-1 is a neuropeptide produced by 50,000–70,000 hypothalamic neurons in the human brain. The HCRT peptides are derived from a precursor, preprohypocretin, which is cleaved into two homologous peptides HCRT1 and HCRT2 [3]. These act on target sites through two receptors, HCRT receptor-1 and HCRT receptor-2. Deficient HCRT neurotransmission is sufficient to produce narcolepsy, as animal models with dysregulated HCRT transmission exhibit a narcolepsy-like phenotype [4], [5]. Of notable interest is a transgenic mouse model where the *HCRT* promoter drives a form of ataxin-3 containing a large polyglutamine repeat, resulting in HCRT cell death and a narcolepsy-like phenotype at 2–3 weeks of age [6].

Human neuropathological studies have extended on these results. *In situ* hybridization (ISH) studies have shown disappearance of *HCRT* mRNA in the perifornical area of narcoleptic brains. Furthermore, the concentrations of HCRT1 and HCRT2 in the cortex and pons, two areas with HCRT projections, are dramatically decreased [7]. Immunohistochemical studies also revealed more than 90% decrease in HCRT cell counts in the hypothalamus of narcoleptic subjects [8]. The loss of HCRT signal is most likely not a simple failure in producing hypocretin peptides alone. Hypocretin cells contain prodynorphin (PDYN) and neuronal pentraxin II (NPTX2; Neuronal Activity-Regulated Pentraxin) [9], [10], and studies have shown that these two proteins are missing in the perifornical area, but not in other regions, of narcoleptic brains [11], [12]. This could imply that HCRT producing cells are quiescent and do not produce HCRT, PDYN and NPTX2 or, more likely, that these cells are missing entirely. Some authors have noted residual gliosis in the perifornical region [13], which combined with the strong HLA association, favors the hypothesis of autoimmune mediated destruction of HCRT neurons. However, most attempts to prove the autoimmune hypothesis, for example through the detection of HCRT-cell specific autoantibodies, have been unsuccessful [14].

A deeper understanding of HCRT cell physiology is required, including the identification of genes and proteins that may be the target of an autoimmune attack or may modulate hypocretin expression/metabolism to make this cell population more susceptible to potential apoptosis. In this study, we have used postmortem human brain samples to compare the transcriptome of narcoleptic versus control subjects, with confirmation in animal models of narcolepsy. Our primary goal was to identify other genes and associated proteins that may be dysregulated in the posterior hypothalamus of narcoleptic patients, potentially expressed in HCRT neurons, as such genes are likely to be novel narcolepsy susceptibility genes.

## Results

### Identification of brain region specific transcripts

A total of 11 control and 6 narcolepsy brains were analyzed by microarray (Table 1). To validate our methods, we first compared transcript abundance across brain regions in control samples and identified brain region specific transcripts (Table S1). Of the 7 identified genes with putative preferential expression in the posterior hypothalamus, three are known to be restricted to this region: prepromelanin concentrating hormone, preprohypocretin, and histidine decarboxylase. Likewise, arginine vasopressin and oxytocin are expressed in the anterior hypothalamus. In the locus coeruleus (LC) where 14 genes were found to be region specific, seven, including dopamine-β-hydroxylase and tyrosine hydroxylase, are known to be expressed specifically in the LC. These results offered a strong validation of sample selection, dissection, array experiment procedure and the statistical analysis methods used in this study. (Table S1)

**Table 1:** Demographic and RNA quality data of human brain tissue used for analysis.

ID	cause of death	age	sex	PMI (h)	brain pH	28S/18S	DQB1	DQB1
C1	not available	77	F	6.0	6.76	0.62	0302	0502
C2	colon cancer	61	M	48.0	6.93	0.9	0301	0301
C5	prostate cancer	77	M	48.0	6.72	0.52	0201	0604
C7	cerebellum cancer meningitis	74	M	4.5	6.62	0.86	0301	0501
C8	kindey cancer; metabolic encephalopathy	75	F	8.0	6.74	0.77	0201	0303
C10	breast cancer	48	F	12.5	6.64	0.64	0503	0603
C12	chronic obstructive pulmonary disease	82	M	5.0	6.78	0.88	0602	0201
C13	dementia	92	M	7.0	6.91	0.65	0602	0602
C14	heart failure	90	M	6.0	6.6	0.53	0602	0602
C15	heart failure	61	F	8.2	6.66	0.5	0602	0301
C16	not available	77	M	2.0	6.63	0.54	0201	0301
N4	not available	n/a	F	n/a	6.63	0.95	0602	0201
N5	lung cancer	68	F	2.5	6.76	1.48	0602	0603
N6	dementia	58	F	42.0	6.55	0.87	0602	0602
N7	dementia	89	F	20.0	6.53	1.12	0602	0201
N8	not available	60	M	3.5	6.7	1.21	0602	0303
N101	epidural hemmorage	69	M	10.5	6.75	0.76	0602	0502

Age and gender distribution, postmortem interval (PMI) and brain pH are not significantly different between narcoleptic subjects (N4–N101) and controls (C1–C16).

### Identification of transcripts dysregulated in human narcolepsy

In the comparison of narcolepsy *vs* control posterior hypothalami, a total of 35 downregulated and 11 upregulated genes were identified by analysis of microarray expression data. Of these, only nine genes were confirmed by Quantitative Reverse Transcriptase-Polymerase Chain Reaction (QRT-PCR), all of which were downregulated in narcolepsy. Hypocretin was the most significantly decreased gene by Significance Analysis of Microarray (SAM) [15] ranking, and was second in terms of mean fold change. QRT-PCR confirmation indicated a dramatic 57.4 fold decrease in preprohypocretin transcript abundance, identifying the known central feature of narcolepsy. The eight other confirmed candidates, leiomodin 1 (LMOD1), cold shock domain protein A (CSDA), G protein-coupled receptor 4 (GPR4), endothelin 1 (EDN1), neuropeptide Y (NPY), growth arrest and DNA-damage-inducible, beta (GADD45B), interleukin 1 recptor-like 1 (IL1RL1) and insulin-like growth factor binding protein 3 (IGFBP3) were downregulated 1.6 to 6.1 fold (Table 2).

**Table 2:** Human narcolepsy susceptibility candidate genes analyzed by microarray, quantitative RT-PCR and distribution shown by *in situ* hybridization in mice.

Microarray data						Quantitative RT-PCR data				*In situ* hybridization data	
Affymetrix Probe ID	Gene Name (Gene Symbol)	Chip	SAM #	1/fold change	P (u-test)	Taqman probe ID	judgement	1/fc	P (u-test)	clone ID	Localization in hyoocretin area
206211_at	selectin E (SELE)	A	3	9.1	0.020	Hs00174057_m1		3.3	0.167	n/a	n.t.
207642_at	hypocretin (HCRT)	A	1	8.7	0.001	Hs00533664_m1	validated	57.4	0.002	6974695	expressed
206346_at	prolactin receptor (PRLR)	A	2	6.1	0.013	Hs00168739_m1		1.0	1.000	5055400	n.c.
203766_s_at	leiomodin1 (smooth muscle) (LMOD1)	A	4	4.5	0.013	Hs00201704_m1	validated	2.2	0.014	3810437	n.e.
238018_at	hypothetical protein LOC285016	B	4	4.4	0.010	Hs01050040_g1		1.3	0.366	5060814	n.e.
235852_at	stonin 2 (STN2)	B	7	4.0	0.010	Hs00263833_m1		1.0	0.584	6475895	n.c.
201161_s_at	cold shock domain protein A (CSDA)	A	7	3.7	0.081	Hs01124963_m1	validated	2.0	0.014	6581631	n.c.
231044_at	RP5-1065J22.5 (LOC127003)	B	8	3.5	0.010	n/a	n.t.			1746040	n.e.
241682_at	kelch-like 23 (KLHL23)	B	77	3.5	0.268	Hs00826289_m1		0.9	0.519	6818752	n.e.
222507_s_at	TMEM9 domain family, member B (TMEM9B)	B	15	3.5	0.106	Hs00221018_m1		0.8	0.465	5347159	n.c.
223333_s_at	angiopoietin-like 4 (ANGPTL4)	B	1	3.5	0.030	Hs00211522_m1		2.6	0.201	5148869	n.e.
226814_at	ADAM metallopeptidase with thrombospondin type 1 motif, 9 (ADAMTS9)	B	6	3.4	0.030	Hs00172025_m1		1.7	0.302	3999494	n.e.
206236_at	G protein-coupled receptor 4 (GPR4)	A	5	3.4	0.001	Hs00270999_s1	validated	1.6	0.053	4038700	n.c.
227697_at	suppressor of cytokine signaling 3 (SOCS3)	B	5	3.2	0.030	Hs00269575_s1		2.1	0.121	6830087	n.c.
217414_x_at	hemoglobin, alpha 2 (HBA2)	A	16	3.2	0.043	Hs00361191_gl		3.0	0.068	5053650	n.e.
206512_at	U2 small nuclear RNA auxillary factor 1-like 1 (U2AF1L1)	A	100	3.2	0.282	Hs00428253_g1		0.9	0.465	6822265	n.c.
230828_at	GRAM domain containing 2 (GRAMD2/LOC196996)	B	13	3.1	0.005	Hs01584657_m1		1.8	0.197	890859	n.e.
241534_at	ATPase, class I, type 8B, member 1 (ATP8B1)	B	91	3.1	0.149	Hs00194444_m1		1.4	0.121	6308651	n.e.
228766_at	CD36 molecule (thrombospondin receptor) (CD36)	B	47	3.0	0.106	Hs00169627_m1		2.1	1.000	3481681	n.e.
211699_x_at	chromosome 11 open reading frame 70 (C11orf70/MGC13040)	B	2	3.0	0.048	Hs00262911_m1		1.6	0.366	6334520	n.e.
222802_at	endothelin 1 (EDN1)	B	25	2.8	0.005	Hs00174961_m1	validated	3.0	0.010	6824438	n.e.
206001_at	neuropeptide Y (NPY)	A	15	2.7	0.020	Hs00173470_m1	validated	3.5	0.039	5683102	n.c.
207896_s_at	deleted in lung and eshophageal cancer 1 (DLEC1)	A	70	2.7	0.181	Hs00201098_m1		1.7	0.366	872087	n.c.
231830_x_at	RAB11 family interacting protein 1 (class I) (RAB11FIP1)	B	19	2.7	0.048	Hs00368787_m1		1.0	0.914	4945175	n.e.
209304_x_at	growht arrestand DNA-damage-inducible, beta (GADD45B)	A	21	2.6	0.005	Hs00169587_m1	validated	2.2	0.020	5032648	n.c.
236894_at	LINE-1 type transposase domain containing 1 (L1TD1/ECAT11)	B	11	2.6	0.034	Hs00219458_m1		undet		962927	n.e.
236034_at	microcephaly, primary autosomal recessive 1 (MCPH1)	B	83	2.6	0.030	Hs00226253_m1		0.8	0.361	6416651	n.e.
203548_s_at	lipoprotein lipase (LPL)	A	68	2.6	0.081	Hs00173425_m1		1.1	0.584	6315514	n.e.
211775_x_at	hypothetical MGC13053	A	41	2.6	0.043	Hs00707696_s1		1.5	0.273	2937689	n.c.
206091_at	matrilin 3 (MATN3)	A	19	2.5	0.020	Hs00159081_m1		4.7	0.302	6306229	n.e.
239151_at	hypothetical protein LOC255326	B	41	2.5	0.015	n/a	n.t.			n/a	n.t.
212143_s_at	insulin-like growth factor binding protein 3 (IGFBP3)	A	11	2.5	0.001	Hs00181211_m1	validated	2.0	0.010	6437611	expressed
207526_s_at	interleukin 1 receptor-like 1 (IL1RL1)	A	40	2.5	0.081	Hs00545033_m1	validated	6.1	0.028	MGC 30073421	n.e.
218775_s_at	WW, C2 and coiled-coil domain containing 2 (WWC2/BOMB)	A	65	2.5	0.181	Hs00227904_m1		1.2	0.273	5142576	n.c.
231728_at	calcyphosine (CAPS)	B	65	2.5	0.268	Hs00362033_g1		1.1	1.000	not expr. in rodents	n.t.

n.t.; not tested, n.e.; no expression, n.c.; no coexpression in perifornical hypocretin area.

probe set ID is from aAffymetrix, QRT-PCR probe ID is from Applied Biosystems, and All clones for in situ hybridization are IMAGE clones except where noted.

Gene expression was compared between 6 narcolepsy and 8 control postmortem posterior hyptothalami (A and B Genechip). Narcolepsy candidate genes selected by statistical analysis of microarray data are listed at left, quantitative RT-PCR results are in the center, and results of *in situ* hybridization in mouse hypothalamus are at the right. As only downregulated genes were confirmed by RT-PCR studies, upregulated candidates are not shown. Genes labeled with “validated” were confirmed as narcolepsy related genes. Note that *IGFBP3* is the only gene validated by QRT-PCR and showing a hypocretin-like distribution pattern. Some candidates showed uniform signal over the whole brain section, potentially indicating ubiquitous expression, but we regarded the staining as background and classified the gene as not expressed if no anatomical variation in staining level was observed.

Previously known HCRT co-expressed genes were not reported in our analysis either because they were not listed within the top 100 SAM ranked candidates (*NPTX2*, *GAL* (galanin), and *CART* (cocaine and amphetamine regulated transcript), or expression was classified as “absent” (*PDYN*) using the microarray suite software (MAS) 5.0 algorithm. The array signals for these genes were decreased (*PDYN*: 2.4 fold, p = 0.005; *NPTX2*: 1.3 fold, p = 0.5; *GAL*: 2.3 fold, p = 0.06; *CART*: 1.2 fold, p = 0.36) as were the QRT-PCR comparisons (*PDYN*: 1.8 fold, p = 0.03; *NPTX2*: 1.6 fold, p = 0.07; *GAL*: 1.9 fold, p = 0.09; *CART*: 1.3 fold, p = 0.24). These results further validated our method.

### IGFBP3 is co-localized in hypocretin producing cells in mouse brains

**Figure 1:**
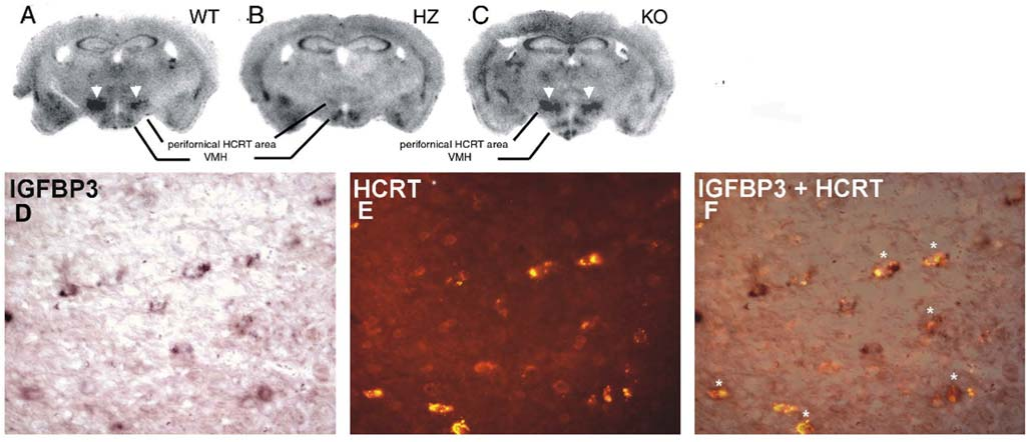
IGFBP3 signals in wild type, ataxin-3 hemizygous, and hypocretin KO mice. The upper panel shows IGFBP3 ISH staining in wild type (A: WT), ataxin-3 hemizygous (B:HZ) and HCRT knockout (C: KO) mice. HCRT staining in neurons (arrowheads) is markedly reduced or absent in the ataxin-3 mouse. The lower panel shows IGFPB3 ISH signal (D: purple; digoxigenin staining with BCIP/NBT), HCRT fluorescence (E: red; Alexa Fluor) immunostaining, and a composite picture (F), indicating that many hypocretin neurons (asterisks) are positive for IGFBP3 in a WT mouse. Scale bar 20 µm.

*In situ* hybridization (ISH) of all human putative downregulated genes was performed in mice (C57/BL/6J) (Table 2). Of the 8 candidates confirmed by QRT-PCR as downregulated in narcolepsy brains, only one gene, Insulin-like Growth Factor Binding Protein-3 (*Igfbp 3*), was clearly enriched in the perifornical region where HCRT neurons are located (Table 2, Fig. 1). Among the other genes, many did not appear to be expressed at all in the studied brain regions or the expression was restricted to regions other than the perifornical area (Table 2).

To determine if IGFPB3 is selectively expressed by HCRT neurons in the perifornical area, we used *Hcrt*-ataxin-3 hemizygous (HZ) transgenic [6], and *Hcrt* knock-out (KO) mice [4]. When we performed ISH with ^35^S-probes and exposed X-ray film to these slides, signal in the perifornical area was visible after 4 day exposure in the littermate wild type (WT) mice, whereas this region remained invisible after 40 days of exposure in ataxin-3 mice (Fig. 1). Loss of HCRT peptide in ataxin-3 mice could potentially induce secondary changes in gene expression in neighboring cells. Therefore we studied *Hcrt* KO mice and found that *Igfbp3* expression was similar to that of WT mice (Fig. 1) indicating that loss of HCRT peptide itself did not induce changes in *Igfbp3* expression.

To investigate coexpression of HCRT and *Igfbp3*, we performed *Igfbp3* ISH followed by HCRT immunostaining. Colocalization of both signals was seen in a majority (∼80%) of hypocretin neurons (Fig. 1). Using this technique, we also surveyed *Igfbp3* expression throughout the mouse brain. Significant *Igfbp3* expression was restricted to a few brain areas including posterior hypothalamus, cerebellar Purkinje neurons, and a group of cells in the pons localized in or close to the pedunculopontine nucleus. Weaker signal was also seen in the ventromedial hypothalamus, granular and pyramidal layers of the hippocampus, and endopiriform nucleus. Expression in all regions other than posterior hypothalamic area was unaffected in the *Hcrt*-ataxin-3 hemizygous (HZ) mice.

### IGFBP3 is localized in human hypocretin producing cells

**Figure 2:**
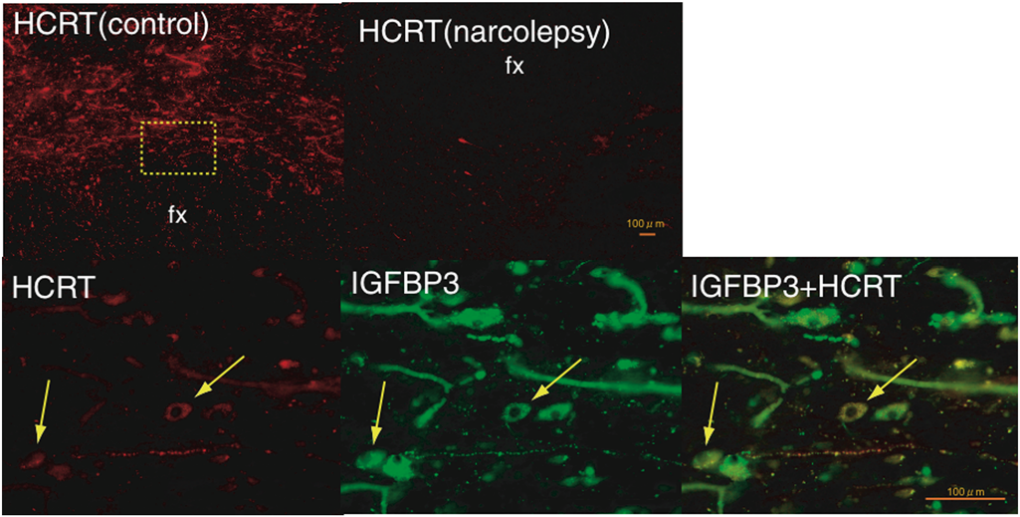
Colocalization of IGFBP3 in HCRT cells in control and narcolepsy human brain. Upper panel: Distribution of hypocretin cells and fibers in the perifornical area of human hypothalamus. (A) In control brains, HCRT cells and fibers were densely stained by an anti-HCRT monoclonal antibody (red fluorescence: VectorRed), while in narcolepsy brains, staining was markedly reduced (B) Lower panel: HCRT immunoreactivity (C: red fluorescence) and IGFBP3 immunoreactivity (D: green fluorescene; Q-dot525) and a composite picture (E) arrows indicate HCRT cells colocalized with IGFBP3). Note non-neuronal autofluorescent elements. Scale bar: 500 µm in A and B, 100 µm in C, D and E.

IGFBP3 immunoreactivity was surveyed using various antibodies (after screening selectivity by western blot) through an entire human hypothalamic block. Blood vessels gave strong signal with IGFBP3 antibody, possibly due to residual blood which has very high IGFBP3 content. Other cells, mostly of non-neuronal origin and negative for the NeuN neuronal marker were also stained. Cellular IGFBP3 staining of NeuN positive large neurons was only noted in the perifornical area. IGFBP3 and HCRT double immunostaining detected colocalization in 10–20% of HCRT neurons in the perifornical area (Fig. 2). All neurons showing colocalization of IGFBP3 and HCRT appeared to have a reduced hypocretin signal. Double-stained fibers were also observed in multiple other hypothalamic areas. This colocalization was also seen in some of the few remaining HCRT neurons in brains from narcolepsy patients.

### Identification of transcripts downregulated in the hypocretin-ataxin-3 hemizygous transgenic mouse model

Gene expression in perifornical posterior hypothalamus was compared in *Hcrt*-ataxin-3 transgenic mice lacking most hypocretin neurons versus wild type animals (two pools of 30 mice). Transcripts with the highest fold changes are reported in Table 3. Genes with known colocalization, such as NPTX2 and dynorphin, were downregulated, validating the model. Other transcripts were also found (Table 3), only one of which, IGFBP3, was downregulated in human narcolepsy hypothalami. For this reason, functional studies involving IGFBP3 and hypocretin were next carried out.

**Table 3:** Mouse transcripts most differentially dysregulated in the perifornical area of ataxin-3 transgenic animals versus control mice.

Affymetrix Probe ID	Gene name (Gene symbol)	Chip	1/Fold change
**Decreased in Hz**			
448821_at	Tyrosinase (Tyr)	A	34.3
452022_at	BAALC isoform 1-6-8 (Baalc)	A	27.9
422411_s_at	Eosinophil-associated ribonuclease 3 (Ear3)	A	16.0
418353_at	CD5 antigen (Cd5)	A	9.2
416128_at	Tubulin, alpha 6 (Tuba6)	A	9.2
426003_at	Neurotrophin-3 receptor non-catalytic isoform 1 (trkC)	A	8.6
451014_at	Receptor tyrosine kinase-like orphan receptor 1 (Ror1)	A	8.0
450772_at	Wingless-related MMTV integration site 11 (Wnt11)	A	7.5
416266_at	Prodynorphin (Pdyn)	A	5.7
418035_a_at	DNA primase, p58 subunit (Prim2)	A	5.3
420471_at	Hypocretin (Hcrt)	A	5.3
439199_at	Protein phosphatase 2a, catalytic subunit, alpha isoform (Ppp2ca)	A	5.3
437618_x_at	G protein-coupled receptor 85 (Gpr85)	A	4.9
421767_at	Adenosine kinase (Adk)	A	4.6
450091_at	Immunoglobulin mu binding protein 2 (Ighmbp2)	A	4.0
427168_a_at	Collagen type XIV (Col14a1)	A	4.0
437502_x_at	CD24a antigen (Cd24a)	A	3.7
432129_a_at	Paired related homeobox 1 (Prrx1)	A	3.7
450315_at	Pheromone receptor V3R8 (V3R8)	A	3.5
455639_at	Rap2 interacting protein (Rap2ip)	A	3.5
423062_at	**Insulin-like growth factor binding protein 3 (Igfbp3)**	A	3.2
435950_at	Periplakin (Ppl)	A	3.2
421516_at	Orphan receptor RTR (Nr6a1)	A	3.2
419628_at	Ceh-10 homeo domain containing homolog (Ch×10)	A	3.0
450533_a_at	Zinc finger protein regulator of apoptosis and cell cycle arrest (Zac1)	A	3.0
427835_at	Transcription factor Oct-1 isoform 7 (Pou2f1)	A	2.8
423026_at	Rad51 homolog c (Rad51c)	A	2.8
425175_at	Gliacolin (C1ql3)	A	2.8
425886_at	ETS-domain transcription factor (Fev)	A	2.6
418494_at	Early B-cell factor 2 (Ebf2)	A	2.6
426180_a_at	MSG2alpha salivary protein (Vcs2)	A	2.6
452380_at	Ephrin receptor A7 (Epha7)	A	2.6
419221_a_at	Regulator of G-protein signaling 14 (Rgs14)	A	2.5
449960_at	Neuronal pentraxin 2 (Nptx2)	A	2.5
421109_at	Camello-like 2 (Cml2)	A	2.5
460354_a_at	Mitochondrial ribosomal protein L13 (L13mt)	A	2.5
427832_at	Testicular alpha tubulin (Tuba-rs1)	A	2.5
**Increased in Hz**			
418162_at	Toll-like receptor 4 (Tlr4)	A	19.7

The perifornical area of 30 wild type and 30 ataxin-3 transgenic mice lacking hypocretin cells were punched (see Fig. S1) and samples pooled to perform microarray experiments. *Hcrt*-ataxin-3 transgenic mouse dysregulated genes are listed by order of transcript abundance (fold change). Most transcripts were decreased in *Hcrt*-ataxin-3 mice, some of which are known to be colocalized with hypocretin (NPTX2, dynorphin). Note that IGFBP3, which was identified by expression profiling using human hypothalami, was also decreased in this experiment.

### Evaluation of IGFBP3 levels, genotype, and IGFBP3 antibodies in blood and CSF of narcoleptic patients

We investigated whether human narcolepsy is associated with dysregulated IGFBP3 levels in the blood and CSF. Mean levels of IGFBP3 in age and sex matched narcolepsy patients (all with low CSF hypocretin-1) versus controls were 59.4±3.4 ng/ml (n = 11) and 58.0±4.5 (n = 11) ng/ml in serum and 31.2±2.1 ng/ml (n = 27) and 29.0±1.9 ng/ml (n = 35) in CSF respectively. As previously reported [16], levels of IGFBP3 correlated with age but not sex in adults. After controlling for these factors, there was no significant difference in age of onset.

A single base polymorphism in the *IGFBP3* promoter region (rs 2854744) strongly correlates with plasma IGFBP3 [17] levels. We tested whether this IGFBP3 polymorphism is associated with human narcolepsy-cataplexy by testing 130 trios using the transmission disequilibrium test (TDT). No difference in transmission was observed (53.5% versus 46.5% for the A allele; Chi sq = 0.77, p = 0.38).

We also explored the possibility that autoantibodies directed against IGFBP3 could be identified. We expressed human IGFBP3 in COS cells, and extracted proteins were western blotted. The resulting membrane was incubated with sera from 22 human narcoleptic and 20 control subjects and revealed with anti human IgG. None of the patients (some with disease onset less than a year prior to blood sampling) or controls had specific reactivity to expressed IGFBP3.

### IGFBP3 inhibits hypocretin production *in vivo*

**Figure 3:**
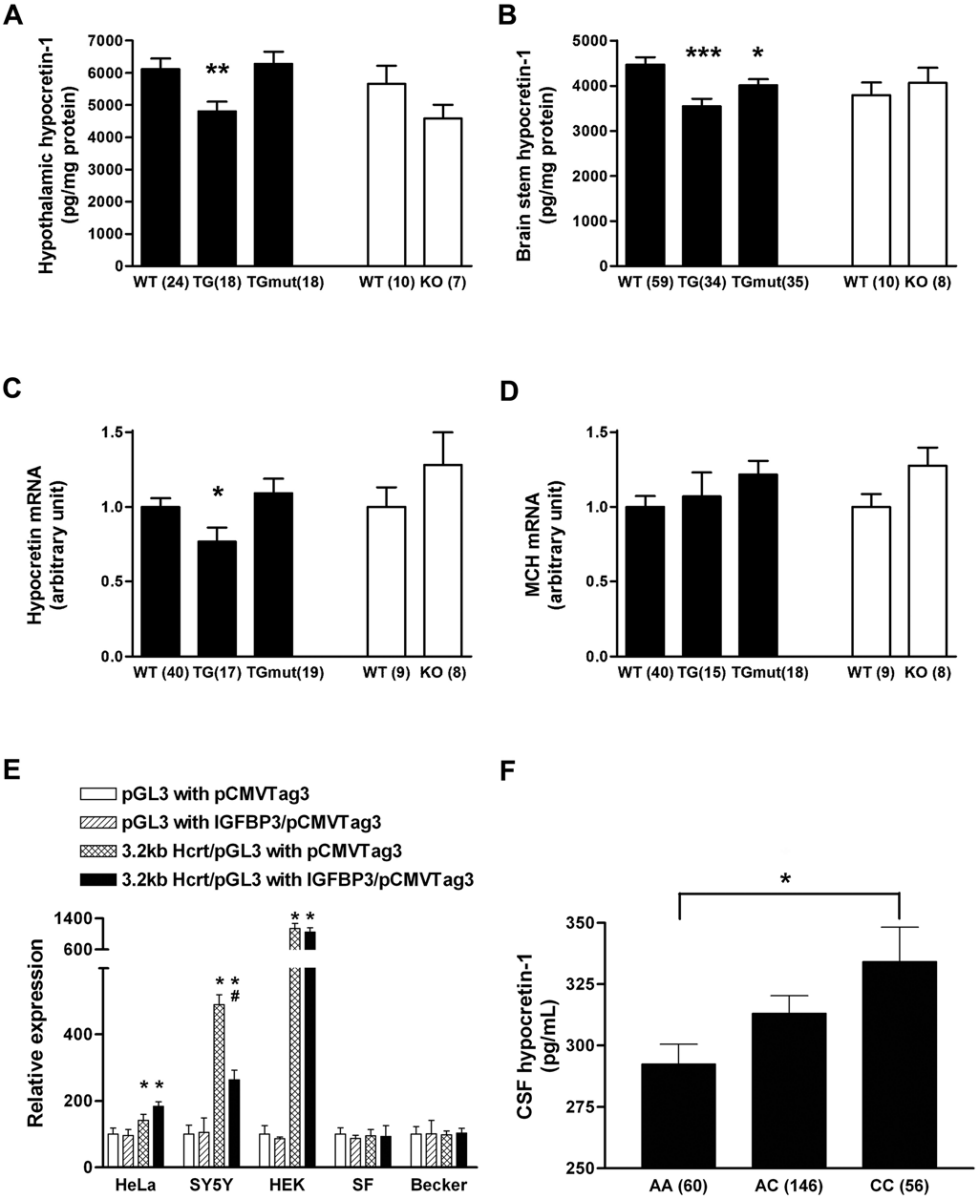
IGFBP3 inhibits HCRT production *in vivo* and *in vitro*. (A, B) Hypocretin-1 peptide content is significantly decreased in both hypothalamus and brainstem of IGFBP3 overexpressing transgenic mice (h*IGFBP3* TG). In mutant IGFBP3 overexpressing mice (hmut*IGFBP3* TG), the hypocretin-1 peptide shows slight but significant decrease only in brainstem, not in hypothalamus (A, B). Hypocretin mRNA is also significantly decreased in IGFBP3 (h*IGFBP3* TG) mice but not in hmut*IGFBP3* TG mice. (C) MCH mRNA level is not affected in h*IGFBP3* TG or hmut*IGFBP3* TG TG mice.(D) IGFBP3 expression reduces preprohypocretin promotor activity in the SH-SY5Y neuroblastoma cell line, but not in non-neural cell lines (HeLa, HEK, SF126, Becker). (F). In human subjects, rs2854744, −202 C, a promotor polymorphism allele known to be associated with reduced IGFBP3 production, is dose dependently associated with increased CSF hypocretin-1 levels. A, B: *: p<0.05; **, p<0.01; ***:p<0.001; C: *: p<0.001 versus without 3.2 kb promotor activity, #:p<0.001 versus promotor activity without IGFBP3; F: *: p<0.05 using ANOVA with genotype as a grouping factor. Sample numbers were indicated in parentheses.

To investigate whether IGFBP3 regulates hypocretin cell physiology and sleep *in vivo*, we studied IGFBP3 knockout mice (*mIgfbp3 KO*: C57BL/6J strain) [18] and two human IGFBP3 transgenic lines ( CD-1 strain) [19]: a transgenic mouse strain overexpressing human IGFBP3 (*hIGFBP3* transgenic), and a transgenic strain overexpressing a mutated form of hIGFBP3 that does not bind IGF (*hmutIGFBP3* transgenic) [20]. These two lines allowed us to distinguish IGF dependent and independent effects of IGFBP3. Quantitative PCR analysis of human IGFBP3 transcripts in the hypothalamus of these models indicated that *IGFBP3* is overexpressed approximately twice in *hIGFBP3* transgenic animals and 10 times in *hmutIGFBP3* transgenic animals, but is absent in IGFBP3 knockout animals (data not shown), validating these models. Immunocytochemistry did not reveal hypocretin cell defects nor decrease in cell number in *Igfbp3* knockout or transgenic animals (data not shown). We next measured hypocretin-1 peptide content and hypothalamic preprohypocretin expression (Fig. 3A–C) and found that preprohypocretin mRNA expression was significantly decreased in *hIGFBP3* transgenic animals compared to controls, but unaltered in the other models (including *hmutIGFBP3* transgenic and *Igfbp3* KO mice)(Fig 3A, C). Hypocretin-1 peptide contents were significantly decreased primarily in *hIGFBP3* transgenic and less so in *humtIGFBP3* transgenic mice compared to controls, indicating both IGF dependent and less prominently independent properties of IGFBP3 affect hypocretin production (Fig 3B). Hypocretin cell counts were nonetheless normal in the *hIGFBP3* transgenic model (3269±99 in HZ versus3230±140 in WT, n = 4 each, age 8 weeks, mean±SEM). As a control, Melanin Concentrating Hormone (MCH) expression was also studied and found to be unaffected by *hIGFBP3* overexpression or *Igfbp3* absence (Fig. 3D).

### IGFBP3 overexpression increases sleep at the end of the active period

**Figure 4:**
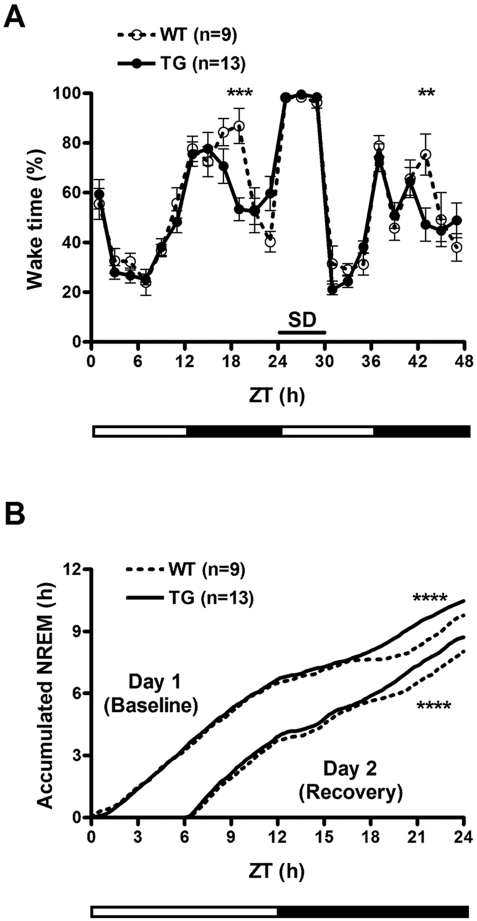
Decreased wakfeluness in human IGFBP3 transgenic mice. (A) Wakefulness is decreased in the second half of the dark/active period in human IGFBP3 transgenic mouse (h*IGFBP3* TG) versus wild type littermates (WT). Reduced sleep rebound is also observed at the same Zeitgeber times (ZT) after sleep deprivation. (B) Cumulative NREM plots at baseline and during recovery after 6 hours of sleep deprivation. Note increased sleep in IGFBP3 transgenic mice in the second half of the active period, ZT17-24. **: p<0.01 versus WT; ***: p<0.001 versus WT. Temperature and locomotion were also affected, see Fig. S2.

To assess whether IGFBP3 overexpression could modulate cell death, *hIGFBP3* transgenic mice were crossed with *Hcrt*-ataxin-3 transgenic mice known to develop hypocretin cell loss at 3–6 weeks of age. Hypocretin cell counts were carried out at 8 weeks of age, and no effect of the *hIGFBP3* transgene was noted (595±36 in double HZ (hemizygous) versus 610±67 in *Hcrt*-ataxin-3/*hIGFBP3* HZ/WT, n = 5 each). We next characterized whether overexpression of IGFBP3 and resulting effects on hypocretin transmission affect sleep and wakefulness by conducting sleep studies on *hIGFBP3* transgenic mice and their littermates. Although no overall significant difference in sleep amounts were observed (Table S2), we found that these animals exhibit more sleep at the end of the dark period. Sleep deprivation was also performed, and revealed a very similar recovery profile, although more sleep was again observed in transgenic animals prior to light onset (Fig. 4). Sleep studies were not performed on *mIgfbp3* KO mice considering the absence of significant differences in Hypocretin mRNA and peptide content in these animals (Fig. 3).

### IGFBP3 reduces promotor activity of hypocretin in neural cell lines

As IGFBP3 is known to have modulatory effects on transcription, for example through its binding to nur 77 [21], a possible explanation for IGFBP3 effects on sleep could be transcriptional modulation of the preprohypocretin gene. To test this hypothesis, prepro-hypocretin promotor activity was examined in several cell lines of various origins in the presence of a transfected IGFBP3 construct (Fig. 3E). We found that IGFBP3 reduced promotor activity of preprohypocretin *in vitro*. This effect was only observed in the neural (neuroblastoma-derived) cell line SH-SY5Y, suggesting the need for neural-specific cofactors.

### An IGFBP3 polymorphism known to increase IGFBP3 serum levels is associated with reduced CSF hypocretin levels

To test whether IGFBP3 activity regulates hypocretin levels *in vivo*, DNA samples of 262 Caucasian subjects with normal CSF hypocretin-1 levels (>200 pg/ml), either drawn from healthy control subjects or from subjects with a complaint of sleepiness unexplained by hypocretin deficiency, were typed for rs2854744 (−202A/C IGFBP3 promoter polymorphism). As previously reported, no relationship between sleepiness/disease status, age or sex and hypocretin levels were found. Interestingly however, a significant dose dependent increase in hypocretin-1 level was found with increasing doses of rs2854744 C, the allele associated with decreased IGFBP3 levels (Fig. 3F). These results suggest that this IGFBP3 polymorphism modulates hypocretin production *in vivo* in humans.

## Discussion

Gene expression profiling in postmortem human brain samples using microarrays is a difficult and controversial area [22]. To validate our technique, we compared transcript abundance in regions known to contain specific neurotransmitters of importance in sleep regulation. After filtering out genes with inconsistent expression, a combination of a permutation method (SAM) and descriptive quantitative ranking of fold changes was found to be the most appropriate statistical analysis. The anterior and posterior hypothalami are known to promote sleep and wake respectively. The LC area was selected as a prototypical HCRT receptor-1 bearing adrenergic cell group, while the diagonal band was selected as an HCRT receptor-2 rich area containing cholinergic neurons.

The comparison of regions showed a remarkably high expression of genes characteristic for these regions (Table S1). In the LC, for example, enriched genes included the presynaptically located α_2A_- receptors and adrenergic transporters, catecholamine synthesizing enzymes (tyrosine hydroxylase, DOPA decarboxylase and dopamine-β-hydroxylase) and the transcription factors *PHOX2A* and *PHOX2B* which are involved in the development of this nucleus [23]. Additional work is indicated to validate and study these genes, as they are potentially important in the neurobiology of these regions. It is notable that our study compared only four regions but was sufficient to effectively identify a large number of region-specific genes.

These promising results led us to compare narcolepsy and control posterior hypothalamus, the primary site of the biochemical defect in narcolepsy (Table 2). In this comparison we used slightly less stringent criteria with inclusion of the top 100 SAM dysregulated genes followed by QRT-PCR validation using the same brain sample sets. No genes were confirmed to be upregulated, whereas 9 of 35 candidates were confirmed as downregulated in the posterior hypothalamus of narcolepsy patients. As expected, preprohypocretin was the top candidate in this analysis, displaying a 9-fold change in expression through the array comparison and a more than 50 fold change when verified by RT-PCR (Table 2). This striking result suggests that if this precise neuroanatomical region had been targeted, it would have been possible to discover the central feature of narcolepsy -HCRT deficiency- without a preconceived hypothesis. A recent microarray analysis in Parkinson's disease also yielded excellent results after careful dissection of the substantia nigra [24]. Our work thus validates the use of postmortem samples for finding the cause of certain neuropsychiatric disorders, a nascent field, provided that careful selection of neuroanatomical regions is performed, as in these studies.

The posterior hypothalamic candidates were also subjected to neuroanatomical screening in mouse brain. The primary goal of this analysis was to identify genes that are coexpressed with HCRT and therefore lost in narcoleptic brains in association with death of these cells. Very few such candidates are currently known, and none is specific for HCRT neurons. Known coexpressed genes in various species include *NTPX2*, *GAL*, *PDYN*, *ENTPD3* (extonucleoside triphosphate diphosphohydrolase 3) and *CART*. These transcripts were not identified in our human array analysis as they either were expressed in other areas of the brain, leading to a low SAM ranking (*NPTX2*, *GAL*, *CART*) or because the expression was classified as absent in most samples (*PDYN*, *ENTPD3*), although the QRT-PCR analysis indicated moderately decreased expression of these genes in narcolepsy (see results).

A combination of QRT-PCR confirmation in human hypothalamus and neuroanatomical screening in the mouse brain identified a single factor, *IGFBP3* that is coexpressed with *HCRT* in both humans and mice and present in only a few other brain areas. Confirmation of the colocalization was performed using multiple techniques in both mouse (Fig. 1) and human (Fig. 2). A combination of ISH and immunostaining showed *Igfbp3* expression in a majority (∼80%) of HCRT neurons in mice. One of the most striking findings was the observation that *Igfbp3* signal decreased dramatically in the perifornical hypocretin cell region of *Hcrt*-ataxin-3 transgenic mouse lacking most hypocretin producing cells.

Although the other 7 QRT-PCR confirmed genes in the human study were not found to be expressed in HCRT neurons, additional work will be needed to explore the importance of these genes in the pathophysiology of narcolepsy. In some cases, expression was not detected by *in situ* hybridization in mice but may still be present at low levels. Further, hypocretin cell loss in narcolepsy might be triggered by yet unknown changes in surrounding cells or structures. These factors would not be identified in animal models with disrupted hypocretin neurotransmission but would be downregulated in human brains. In this respect, *IL1RL1* may be of special interest as we found a 6.1 fold difference by QRT-PCR, and this orphan receptor has a role in immune regulation [25]. The other downregulated genes that were found, or previously known to express elsewhere (*eg NPY*, *GPR4*), may reflect physiologically important network remodeling in narcolepsy.

Double immunostaining also revealed additional IGFBP3 immunoreactive, hypocretin negative cells in human hypothalamus (both neurons and glial cells). The IGFBP3 protein is primarily produced and secreted by the liver and is the major carrier of insulin-like growth factors 1 and 2 (IGF1, IGF2) in the blood. The resulting IGFBP3-IGF complex is typically taken up by cells through IGF receptor 1 to produce intracellular effects [26]. In this context, uptake of IGFBP3 by brain cells could render them immunoreactive without accompanying gene expression. We found that CSF levels of IGFBP3 were high, suggesting significant translocation from serum into brain extracellular fluid. Other explanations could involve antibody specificity issues and relative differences in abundance between protein and mRNA in different cells. Induction of *IGFBP3* expression can occur in neurons and glial cells under various conditions, for example hypoxia [27], and this might also cause differences between humans and mice.

What function could IGFBP3 have in hypocretin producing cells with regard to narcolepsy? As the protein is co-localized with HCRT, IGFBP3 could have been an autoantigen involved in the hypothesized autoimmune attack directed against the HCRT neurons. We found no evidence for such autoantibodies in human sera and CSF, however. IGFBP3 levels were similar in CSF and serum of narcolepsy versus controls, and it is abundant in various human brain cells, possibly reflecting cellular uptake of circulating IGFBP3. These make it an unlikely candidate in directing an autoimmune process specifically toward HCRT neurons.

Even if not directly involved as an autoantigen in causing narcolepsy, IGFBP3 is still an interesting candidate as it plays key roles in regulating cell proliferation and apoptosis [28]. The interaction of IGFBP3 with IGFs is generally believed to have primarily pro-growth effects by favoring target availability of IGF, but growth-inhibiting effects have also been noted. IGFBP3 is also reported to have proapoptotic properties independent of IGF-binding. For example, IGFBP3 expression in cancer cells and in the circulation is associated with less malignant growth and decreased cancer risk in breast [29], prostate [30] and other cancers [31]. Of note, the proapoptotic effects commence with IGFBP3 translocation into the nucleus and binding of the retinoid-X-receptor-α (RXRα), with subsequent mobilization of the RXRα binding partner Nur77 from the nucleus to mitochondria, an event followed by caspase activation and apoptosis [21]. We found no intranuclear IGFBP3 staining in human hypocretin cells (Fig 2 D,E), suggesting no proapoptotic effects in surviving hypocretin cells.

Recently however, proapoptotic effects of IGFBP3 independent of nuclear translocation and protein secretion have been demonstrated, suggesting additional cytoplasmic pathways also promote apoptosis [30]. It is thus possible that IGFBP3 produced within hypocretin cells would not be bound to IGFs, potentially increasing vulnerability to proapoptotic processes. Indeed, hypocretin neurons in slice cultures are more sensitive than neighboring cells to NMDA receptor-mediated injury [32]. To test the hypothesis that excessive IGFBP3 in hypocretin cells contributes to this cell death, we crossed *Hcrt*-ataxin-3 transgenic mice (animals with targeted cell death 2–4 weeks after birth) with transgenic mice overexpressing human IGFBP3 (*hIGFBP3*), but found no effects on the speed of hypocretin cell death. The lack of effects of IGFBP3 on cell death in this model might be due to the differences in circulating *vs* hypocretin cell specific IGFBP3 overexpression, or to lack of an IGFBP3 effect in the context of ataxin-induced cell degeneration, but is consistent with the notion that IGFBP3 may be proapoptotic only in specific circumstances, such as cancerous cells.

The results above do not support the involvement of IGFBP3 in causing hypocretin cell death in narcolepsy. We therefore next explored whether IGFBP3 modulates hypocretin transmission, with complementary *in vitro* and *in vivo* functional studies using *hIGFBP3* transgenic mice. We found that increased IGFBP3 decreases both hypocretin mRNA, and hypocretin peptide content in hypothalamus and target areas (Fig. 3A–C). Although these effects could be indirectly mediated by minor endocrine abnormalities in these animals, for example hyperglycemia [33], the *in vitro* findings that IGFBP3 expression suppresses *HCRT* promotor activity (Fig. 3E) and that the functional IGFBP3 polymorphism rs2854744 is associated with reduced hypocretin transmission, observed as lower levels of Hcrt-1 in human CSF (Fig. 3F), makes this hypothesis unlikely. Overexpression of *hmutIGFBP3*, a mutant form that does not bind IGF, also reduces hypocretin peptide content in brainstem but not in hypothalamus suggesting both IGF dependent and independent effects on hypocretin transmission. Hypocretin transmission was normal in *Igfbp3* knockout mice, potentially reflecting functional redundancy among the 7 known IGFBP family members.

Increased IGFBP3 expression (which was stable across the 24 hrs) was shown to have functional effects on sleep, as *hIGFBP3* transgenic mice slept significantly more prior to light onset. It is notable that the effect was primarily observed at the end of the active period. As hypocretin release is highest at this time of the day (equivalent to the evening in humans), higher IGFBP3 levels may affect hypocretin transmission only at times of highest demand, through the reduction of releasable peptide stores in terminals.

IGFBP3 and IGF serum levels are highest around puberty, and drop thereafter [16], [34]. It is thus interesting to speculate that increased sleepiness during puberty could coincide with peak IGFBP3 levels. Most work to date has focused on the studies of Growth Hormone, the primary determinant of IGF, while the effects of IGF on sleep seem complex [35], [36]. Additional investigations of the chronic effects of IGFs and IGFBP3 on sleep changes around puberty are needed [34]. IGFBP3, together with IGF, is to be added to the growing list of metabolic indicators that have been reported to regulate hypocretin activity.

In summary, this study exemplifies the successful use of human postmortem brain for microarray analysis of human neuropathology. The analysis not only confirmed known genes colocalized with hypocretin cells but also identified a new candidate with functional relevance to hypocretin cell physiology and sleep regulation. This factor, IGFBP3, is only expressed at high levels in a few neuronal cell groups besides hypocretin cells, and regulates hypocretin transcription. We hypothesize that increased IGFBP3 amount in HCRT cells decreases HCRT production and reduces wake under physiological conditions. When expression exceeds a specific threshold however, it may initiate hypocretin cell death and cause narcolepsy.

## Materials and Methods

### Human studies

#### Human samples

All blood and DNA samples were of Caucasian origin. Narcolepsy and control brain donors were primarily recruited through the Stanford narcolepsy brain donation program and the Stanford Brain Bank. Samples from 9 narcoleptic patients (89% Caucasian) and 14 Caucasian controls were dissected. Six narcolepsy and 11 control samples were analyzed after passing quality control (Table 1). Patients were all HLA DQB1*0602 positive with cataplexy. Sera from 11 narcolepsy and 11 controls, CSF from 27 narcolepsy and 35 controls were also used for IGFBP3 measurements. DNA samples from 130 parent-child trios (proband and parents), and 252 individuals with available CSF hypocretin level values in the control range, were obtained and used. Informed consent was obtained in accordance with Stanford human subjects policy and the principles of the Declaration of Helsinki.

#### Brain dissection

Four brain regions were dissected: posterior and anterior hypothalamus, LC and diagonal band of Broca. Coronal sections (0.9 mm) of hypothalamus and diagonal band, and transaxial brainstem sections were cut from frozen blocks, mounted, and stored at −80°C. Digital photographs of the blocks were used for orientation and identification of target regions. The location of hypothalamic structures and the diagonal band were determined using human atlas coordinates [37]. The LC was identified by atlas location [38] and coloration.

Posterior hypothalamic samples were collected from the mammillary body (atlas fig.32, optic chiasm +13 mm) to the level where the fornix enters the hypothalamus (fig. 25, optic chiasm +4 mm). Anterior hypothalamic samples were collected from this level (fig. 24, optic chiasm +3 mm) to the optic chiasm (fig. 19, optic chiasm −2 mm). Diagonal band areas closely surrounded the anterior hypothalamus in the same planes. Hypothalamic and diagonal band samples were dissected by scalpel, LC samples were collected with a 1.2 mm Palkovits punch (Stoelting Co., Wood Dale, IL).

#### RNA isolation and Array Hybridization

Biotinylated cDNA synthesized from total RNA was hybridized to microarrays (HG-U133 A and B, Affymetrix, Santa Clara, CA) according to manufacturer protocols (Genechip manual, Affymetrix). Fluorescent array images were scanned (Affymetrix GeneArray 2500 or GeneChip 3000 scanner) and analyzed with global scaling, adjusting mean target intensity to 500 for all probe sets (Affymetrix MAS 5.0 software).

#### Quality control and sample comparisons

The pH of each sample was measured (homogenate of a 10× dilution of 0.5 g of temporal cortex or striatum in water). Samples with pH≤6.5 were excluded [39]. Integrity of extracted RNA was verified by RNA nano LabChips on a 2100 Bioanalyzer (Agilent Technologies, Palo Alto, CA). Samples with a 28S/18S ratio below 0.5 were discarded (median ratio 0.81). Microarray data was also used to estimate RNA quality (ratio of *GAPDH* 3′ over 5′ probe hybridization). Samples with a ratio over 8 were excluded (median ratio 2.3). Postmortem interval had less effect on RNA quality and subjects were included independent of interval (median 7 hrs, all below 48 hrs).

We compared regional transcript abundance in 11 control brains (74.0±13.0 years old, PMI: 14.1±17.0 hr, 63.6% male, 36.4% HLA DQB1*0602 positive). Sample size for brain regions (varied due to availability and sample quality) included 8 posterior hypothalami, 6 anterior hypothalami, 7 diagonal bands and 4 LCs. We compared posterior hypothalami of 6 narcoleptic patients (68.8±12.3 years old, PMI 15.7±16.3 hr, 33.3% male, all DQB1*0602 positive) and 8 controls (73.0±13.0 years old, PMI 11.8±15.0 hr, 62.5% male, 37.5% DQB1*0602 positive) for the A chip, and 5 narcolepsy and 7 control samples for the B chip (Table 1). Overall mean age, PMI and sex did not differ significantly.

#### Statistical analysis of array data

MAS 5.0 was used for signal calculation and present/absent determination combined with SAM ranking analysis to identify significantly up/down regulated genes. Data from the A and B chips were analyzed separately. Probes with an absent or marginal call in more than 90% of samples were omitted as were array tags linked to multiple genes, and only the highest SAM ranked probe for a gene was included. Filtering eliminated 41% and 53% of the transcripts from the A and B chips in the narcolepsy *vs* control comparison. Results were reported as SAM rankings, fold changes in average expression levels, and Mann-Whitney U-test *p*-values. The top 15 SAM upregulated genes were used for comparisons across brain regions. Genes upregulated in two of three comparisons were reported. For the comparison of posterior hypothalamus between narcolepsy and control, the top 100 SAM up or down regulated candidates were selected and sorted based on fold changes: those above 2.5 or below 0.4 fold change were studied further.

#### Quantitative Reverse Transcriptase PCR

Candidate transcripts were studied by QRT-PCR (ABI 7300 system, Applied Biosystems, Foster City, CA). cDNA was synthesized from total RNA using Superscript III Reverse Transcriptase and random hexamer.(Invitrogen, Carlsbad, CA). Geometric means of β-actin and β2-microglobulin were used for normalization (geNorm analysis) [40]. After performing QRT-PCR for selected genes in parallel with β-actin and β2-microglobulin, relative expression quantity was calculated. Genes were considered validated when the mean fold change was more than 1.5 and Mann-Whitney U-test indicated statistical significance (p<0.05). Expression differences for *CART* [41], *GAL* [42], *PDYN* [9] and *NPTX2* [10] were also verified.

#### Human brain immunohistochemistry

Six hypothalami (4 control, 2 narcolepsy) were fixed in 4% paraformaldehyde (PFA, pH 7.3), cryoprotected and sectioned coronally to obtain a series of 24 sections (40 µm). The following steps were performed at 4°C interspersed with washes. Sections were (I) treated with 0.3% H_2_O_2_, (II) post-fixed with 4% PFA, (III) blocked in 1.5% horse serum, (IV) incubated with mouse anti-HCRT monoclonal antibody (1∶250) [43] or a monoclonal anti-NeuN antibody (1∶50,000;Millipore, Billerica, MA) (V) incubated with biotinylated horse anti-mouse IgG (1∶200; Vector Laboratories, Burlingame, CA), (VI) alkaline phosphatase (AP) conjugated ABC (1∶100; Vector), and (VII) VectaRed AP-substrate (1∶50; Vector) in 0.1 M Tris-HCl (pH 8.4) until satisfactory staining was obtained. Sections were then (VIII) incubated in 1.5% rabbit serum, (IX) goat anti-IGFBP3 antiserum (1∶250; AF675 R&D Systems, Minneapolis, MN), (X) biotinylated rabbit anti-goat IgG (1∶200, Vector), (XI) ABC reagent (1∶100; Vector), (XII) biotinyl-tyramide (1∶500; PerkinElmer) with 0.03% H_2_O_2_, and (XIII) Qdot 525 streptavidin conjugate (1∶100;Invitrogen) in borate buffer (pH 8.5). Sections were mounted and analyzed under a fluorescence microscope equipped with a CCD camera: images were digitally merged to visualize the colocalization of signals.

#### Evaluation of hypocretin and IGFBP3 levels, antibodies and IGFBP3 genotyping

CSF and serum IGFBP3 levels were measured in duplicate using a total ELISA kit (DSL-10-6600; Diagnostic Systems Laboratories, Webster, TX) according to the manufacturer's protocol. Average intra-assay coefficients of variation were 2.5%. CSF hypocretin-1 levels were measured using a radioimmunoassay as reported previously [2]. We tested CSF of 27 narcolepsy and 35 matched controls, and serum from 11 narcolepsy and 11 controls.

Full length *IGFBP3* cDNA (EcoR1-ApaI fragment, clone 5287665, Invitrogen) was subcloned into pCMV-Tag3 and transfected into COS-1 cells (Lipofectamine 2000, Invitrogen). Protein was extracted from cells and culture medium by standard methods (RIPA buffer, and protocol Sigma, St. Louis, MO).

Protein extracts were run on 10% SDS-PAGE gels, transferred onto nitrocellulose, and incubated with anti-human IGFBP3 polyclonal antiserum (1∶200; Santa Cruz Biotechnology, Santa Cruz, CA), followed by horseradish peroxidase (HRP)-conjugated donkey anti-goat IgG (1∶4000) and then detected with Supersignal West Pico chemiluminescence reagent (ThermoFisher, Wyman, MA), revealing the 42 kDa IGFBP3 band. Similar membranes were used to detect anti IGFBP3 antibodies in human sera. Membranes were incubated with serum (1∶200) followed by HRP-donkey anti-human antiserum (1∶5000), and chemiluminescent detection. Twenty two narcolepsy and 20 control samples were tested.

The single base pair polymorphism in the *IGFBP3* promoter (rs2854744, −202 A/C) was genotyped in 130 Caucasian narcolepsy-cataplexy trios and 262 adult Caucasian subjects (54% females; mean age 35.8±0.8 years) using established methods [17].

### Mouse studies

#### Animals and tissue preparation

Five different mouse lines were used: hypocretin-ataxin-3 transgenic mice lacking hypocretin neurons (*Hcrt*-ataxin-3: C57BL/6J background), preprohypocretin knockout mice (*Hcrt* KO: C57BL/6J background), IGFBP3 knockout mice (*mIgfbp3* KO: C57BL/6J background) (courtesy of Dr JE Pintar, University of Medicine and Dentistry of New Jersey, Piscataway, NJ) [18], transgenic mice overexpressing human IGFBP3 cDNA (*hIGFBP3* transgenic: CD1 background), transgenic mice overexpressing the Gly56/Gly80/Gly81 mutated form of human IGFBP3 which lacks IGF binding (*hmutIGFBP3* transgenic: CD1 background) (courtesy of Dr. LJ Murphy, University of Manitoba, Winnipeg, Canada) [19]. The latter two mouse strains allow differentiation between IGF-bound and IGF-independent effects of the protein. Two human *IGFBP3* genes were driven by the same mouse phosphoglycerate kinase I promotor, and were used to distinguish the effect of IGF binding. In all transgenic comparisons, age matched mice of the corresponding genetic background, usually littermates, were used at all times. Mice were maintained under controlled temperature (21±1°C) and 12 h:12 h light-dark cycle with free access to food and water. The entire study was approved and conducted in accordance with the guidelines of Stanford's Administrative Panel for Laboratory Animal Care.

For neuroanatomical studies, mice were euthanized (pentobarbital) and perfused transcardially with saline followed by 50 ml 10% formalin (pH 7.4). Brains were fixed in 10% formalin, and equilibrated with 20% sucrose/0.5% formalin. Coronal slices (30 µm) containing the whole hypothalamus were sectioned into a 1∶5 series and mounted.

#### Mouse microarray experiments

Groups of 30 wild type and 30 ataxin-3 transgenic mice were used. Perifornical hypothalamic 0.5 mm Palkovits punches encompassing the hypocretin field (Fig. S1) were collected at ZT 22. Biotinylated cRNA was synthesized from total RNA and hybridized to Affymetrix Mouse 430 microarrays and scanned fluorescent array images were analyzed with GeneChip Operating Software (Affymetrix)

#### *In situ* hybridization for candidate genes

*In situ* hybridization was performed on 6–10 week male C57BL/6J mice. Mouse cDNA IMAGE clones (Table 2; Invitrogen) were sequence verified (Bionexus, Oakland, CA) and used. Plasmid DNA was linearized and transcribed with T7, T3 or SP6 polymerases (Promega, Madison, WI) and ^35^S-UTP (Amersham BioSciences, Piscataway, NJ) or digoxigenin-UTP (Roche Diagnostics, Indianapolis, IN) by standard methods.

Probes were diluted in standard hybridization buffer to 3×10^6^ counts per 125 µl. Sections were pretreated in citrate buffer (pH 6.0), and hybridized with probe at 54°C, followed by RNase A treatment and stringent washes (2×SSC at 50°C; 0.2×SSC, 55°C; 0.2×SSC, 65°C), dehydrated and exposed to films for 1–40 days.

#### In situ hybridization of IGFBP3 and HCRT immunostaining

Digoxigenin-labeled probe (1∶500) was used in hybridizations as described. Sections were treated with 3% sheep serum/0.1% Triton X-100, and incubated overnight with alkaline phosphatase-conjugated sheep anti-digoxigenin antibodies (1∶5000; Roche). Endogenous alkaline phosphatase was blocked (levamisole) and hybridization was visualized by incubation in 0.3 mg/ml NBT(nitroblue tetrazolium) and 0.2 mg/ml BCIP(5-bromo,4-choloro,3-indolylphosphate).

Sections with satisfactory *IGFBP3* signal were immunostained with highly specific rabbit anti-HCRT-1 antiserum (1∶4000; made with human HCRT-1 as immunogen). Slides were washed and incubated with (I) biotinylated goat anti-rabbit IgG (1∶500; Jackson Immunoresearch, West Grove, PA), (II) ABC complex (1∶1000; Vector), (III) biotinylated tyramide diluted 1∶50 in amplification buffer (Perkin Elmer), (IV) Alexa Fluor-conjugated streptavidin (1∶200;Invitrogen).

To stain and count hypocretin cell populations, successive sections encompassing the entire hypocretin field were stained using an anti HCRT-1 antiserum as described above. Cells were counted without corrections and blind of genotype status.

#### Hypocretin-1 radioimmunoassay

Frozen brain tissue of animals sacrificed at Zeitbeger time ZT2–ZT3 were extracted with 1 mL of 0.5 M acetic acid and boiled in water bath for 15 minutes. Samples were cooled on ice and centrifuged at 5000×g for 10 minutes. Protein concentration in the supernatant was measured using the Bradford method (Bio-Rad Laboratories, Hercules, CA). The supernatants were dried overnight at 50°C and reconstituted in RIA buffer for radioimmunoassay using a commercially available ^125^I RIA kit (Phoenix Pharmaceuticals, Belmont, CA) as described [44]. The hypocretin contents were corrected against protein concentrations.

#### Preprohypocretin, murine/human IGFBP3 and MCH mRNA quantification

Total RNA from mice hypothalamic regions with RNA extraction reagents (Qiagen, Valencia, CA) and synthesized cDNA was subjected to TaqMan real time PCR analysis to measure relative preprohypocretin, murine/human IGFBP3, and MCH expression levels in parallel with ß-actin, hypoxanthine-guanine phosphoribosyltransferase (HPRT) and GAPDH as internal controls. HPRT was chosen for data normalization due to its stable expression across the genotypes.

#### Mouse sleep recording and analysis

Nine wild type (WT) and 13 transgenic (TG) mice (age 3–6 months) were implanted under isofluorane anesthesia with telemetry transmitters (ETA-F20, 3.9 g weight, Data Science International, St. Paul, MN) capable of acquiring and sending electroencephalograph (EEG), temperature, and movement data. The two EEG electrodes were secured with dental cement at the following coordinates: anterior/posterior from bregma (AP) 1.5 mm, lateral (ML) 1.5 mm and AP −3.5 mm, ML −3 mm. An analgesic (5 mg/kg Carprofen) and antibiotics (5 mg/kg/day enrofloxacin) was given subcutaneously. Mice were allowed to fully recover for a minimum of two weeks before the experiments. Animals were recorded for a 48-hour period, with the first 24 h undisturbed, followed by 6 h wake extension by gentle handling, and 18 h undisturbed recovery. EEG was sampled at 250 Hz, and the other parameters were sampled at 50 Hz using DataQuest A.R.T. 3.1 (Data Science International, St. Paul, MN). Recordings were scored manually in 10 second epochs using SleepSign (Kissei America, Irvine, CA) according to the method developed previously [45].

### Hypocretin promoter studies

#### Cell culture

HeLa (human cervical carcinoma), SF126 (human glioblastoma), and Becker (human astrocytoma) cells were grown in Dulbecco's modified Eagle's medium (DMEM; GIBCO, Grand Island, NY) supplemented with 10% fetal bovine serum (GIBCO),.and SH-SY5Y (human neuroblastoma) cells were grown in DMEM/F12 supplemented with 5% horse serum (GIBCO), at 37°C and 5% CO_2_.

#### Reporter plasmids

The pGL3-basic plasmid (Promega) encoding the firefly luciferase gene was used for the promoter activity assessment with introduced sequence and the pRL-TK plasmid (Promega) encoding Renilla luciferase was used as internal control for transfection efficiency. The plasmid 3.2 kb Hcrt/pGL3 was constructed by cloning the HCRT promoter sequence at −3278/+87 [46] into upstream of the firefly luciferase gene in pGL3-Basic plasmid; 5′- ccgctcgagGGTGTCTGGCGCTCAGGGTG-3′ (corresponds to the first exon sequence just before the translation initiator ATG of human prepro-Hcrt gene, and 5′- CGACGCGTGGATCCAGATGCCTCTGAATAG-3′ (−3278) were used.

#### Transient transfection

Cells were seeded at a 250,000 per well in 24-well cell culture plate coated with collagen type I (BD Biosciences, Bedford MA) one day before transfection. Cells were co-transfected with three types of plasmid mixed in the following amount per well with FuGENE 6 Transfection Reagent (Roche): 200 ng firefly luciferase-encoding reporter plasmid (pGL3-basic or 3.2 kb Hcrt/pGL3), 20 ng Renilla luciferase-encoding internal control reporter plasmid (pRL-TK), and 200 ng expression vector (pCMV-Tag3 as mock or IGFBP3/pCMV-Tag3).

#### Luciferase activity measurements

At 24 h after transfection, cells were washed and lysed with 100 µL passive lysis buffer (Promega). Activities of two luciferases encoded by reporter plasmids and internal control plasmids were measured sequentially twice using the Dual-Luciferase Reporter assay reagents (Promega) and PLATE CHAMELEON multilabel platereader (HIDEX, Finland) according to the manufacturer's protocol. Relative luciferase activity (RLA) was determined by FLU value divided by RLU value. All RLA values were further standardized by the reference RLA value for pGL3-basic plasmid with pCMV-Tag3 vector (mock) as 1.0.

## Supporting Information

Table S1 Cross-regional comparison of transcript abundance in selected human brain regions. Comparison of microarray expression data across 4 brain regions in control subjects The listed genes have enriched expression in the indicated region, as compared to the other regions. For example, PMCH gene expression was increased 73.8 fold in the posterior hypothalamus when compared to the anterior hypothalamus and ranked #1 using the SAM analysis. Similarly, PMCH gene expression in the posterior hypothalamus ranked first versus the diagonal band sample (3.3 fold change), and the LC (275.1 fold change). Only genes enriched in one region versus at least two other regions are listed (see methods).
(0.13 MB DOC)

Table S2 Overall sleep parameters in mice overexpressing human IGFBP3 (TG) versus wild type littermates (WT)Although total wake is decreased in TG mice, the difference is not statistically significant. Hour by hour analysis revealed that decreased wake occurred prior to light onset (see Fig. 4).
(0.06 MB DOC)

Figures S1 Perifornical hypothalamic region dissected for microarray analysis in mice (A) and transcript abundance distribution plots correlating abundance in wild type versus ataxin-3 transgenic mice (B). (A) Immunocytochemistry of hypocretin in wild type (WT, top) and Hcrt-ataxin-3 transgenic mouse (bottom). The area collected using punches is outlined by a circle in wild type versus transgenic mice lacking most hypocretin cells. Transcript abundance distribution (B) in both genotypes is highly correlated, and hypocretin is one of the outliers (circled dot). For list of differentially regulated transcripts, see Table 3.
(1.56 MB TIF)

Figure S2 Locomotion (A) and temperature (B) in wild type (WT) versus hIGFBP3 transgenic mice (Tg). Note decreased temperature and locomotion at the end of the active period in hIGFBP3 transgenic mice, mirroring changes in sleep depicted in Fig. 4.
(5.70 MB TIF)
