# Supplementary material for: IGFBP3 Colocalizes with and Regulates Hypocretin (Orexin)
Source: PLoS One. 2009 Jan 22;4(1):e4254. doi: 10.1371/journal.pone.0004254 (PMC2617764; doi:10.1371/journal.pone.0004254)
Supplement: Table S1 — Cross-regional comparison of transcript abundance in selected human brain regions. Comparison of microarray expression data across 4 brain regions in control subjects The listed genes have enriched expression in the indicated region, as compared to the other regions. For example, PMCH gene expression was increased 73.8 fold in the posterior hypothalamus when compared to the anterior hypothalamus and ranked #1 using the SAM analysis. Similarly, PMCH gene expression in the posterior hypothalamus ranked first versus the diagonal band sample (3.3 fold change), and the LC (275.1 fold change). Only genes enriched in one region versus at least two other regions are listed (see methods). (0.13 MB DOC) [file pone.0004254.s001.doc]

| **Supplementary Table 1: Cross-regional comparison of transcript abundance in selected human brain regions** | | | | | | | | | | | | | | | | | |
| --- | --- | --- | --- | --- | --- | --- | --- | --- | --- | --- | --- | --- | --- | --- | --- | --- | --- |
|  |  |  | **Posterior Hypothalamus** | | |  | **Anterior Hypothalamus** | | |  | **Diagnal Band** | | |  | **Locus Coeruleus** | | |
|  | **Gene Name** (gene symbol) |  | fold change | SAM# | p(u-test) |  | fold change | SAM# | p(u-test) |  | fold change | SAM# | p(u-test) |  | fold change | SAM# | p(u-test) |
| **Posterior Hypothalamus** | pro-melanin-concentrating hormone (PMCH) |  | 1.0 |  |  |  | 73.8 | 1 | 0.001 |  | 3.3 | 1 | 0.021 |  | 275.1 | 1 | 0.004 |
|  | pro-melanin-concentrating hormone-like 1 (PMCHL1) | | 1.0 |  |  |  | 36.9 | 2 | 0.001 |  | 3.5 | 2 | 0.021 |  | 19.3 | 6 | 0.004 |
|  | parvalbumin (PVALB) |  | 1.0 |  |  |  | 21.2 | 4 | 0.001 |  | 6.8 | 6 | 0.014 |  |  |  |  |
|  | histidiine decarboxylase (HDC) |  | 1.0 |  |  |  | 12.8 | 3 | 0.001 |  | 3.2 | 3 | 0.014 |  | 24.0 | 5 | 0.004 |
|  | hypocretin neuropeptide precursor (HCRT) |  | 1.0 |  |  |  | 5.3 | 6 | 0.005 |  | 6.1 | 5 | 0.002 |  | 12.7 | 15 | 0.004 |
|  | solute carrier family 35, member D3 (SLC35D3) |  | 1.0 |  |  |  | 4.2 | 1 | 0.014 |  | 2.0 | 73 | 0.073 |  | 10.8 | 4 | 0.017 |
|  | synaptogtagmin VI (SYT6) |  | 1.0 |  |  |  | 3.9 | 5 | 0.001 |  | 2.3 | 58 | 0.011 |  | 5.0 | 12 | 0.017 |
| **Anterior hypothalamus** | arginine vasopressin (AVP) |  | 2.1 | 28 | 0.142 |  | 1.0 |  |  |  | 3.1 | 1 | 0.035 |  | 315.7 | 1 | 0.010 |
|  | oxytocin (OXT) |  | 1.6 | 633 | 0.345 |  | 1.0 |  |  |  | 3.0 | 2 | 0.035 |  | 206.0 | 3 | 0.010 |
|  | forkhead box G1B (FOXG1B) |  | 5.2 | 1 | 0.001 |  | 1.0 |  |  |  | 1.2 | 855 | 0.731 |  | 167.3 | 2 | 0.010 |
|  | dendrin (DDN) |  | 9.2 | 2 | 0.020 |  | 1.0 |  |  |  | 1.2 | 472 | 0.628 |  | 20.7 | 14 | 0.019 |
|  | LIM homeobox2 (LHX2) |  | 5.0 | 3 | 0.001 |  | 1.0 |  |  |  | 1.3 | 200 | 0.445 |  | 17.9 | 6 | 0.010 |
|  | centaurin, gamma1 (CENTG1) |  | 3.7 | 7 | 0.051 |  | 1.0 |  |  |  | 0.8 | 445 | 0.445 |  | 13.5 | 7 | 0.024 |
|  | secretagogin, EF-hand calcium binding protein (SCGN) | | 1.7 | 930 | 0.755 |  | 1.0 |  |  |  | 3.1 | 6 | 0.051 |  | 11.8 | 13 | 0.010 |
| **Diagonal Band** | forkhead box G1B (FOXG1B) |  | 4.4 | 1 | 0.009 |  |  |  |  |  | 1.0 |  |  |  | 141.5 | 1 | 0.006 |
|  | dendrin (DDN) |  | 7.7 | 2 | 0.094 |  |  |  |  |  | 1.0 |  |  |  | 17.3 | 15 | 0.073 |
|  | LIM homeobox2 (LHX2) |  | 3.9 | 14 | 0.072 |  |  |  |  |  | 1.0 |  |  |  | 13.9 | 9 | 0.006 |
|  | B-cell CLL/lymphoma 11B (BCL11B) |  | 5.6 | 10 | 0.073 |  | 2.5 | 85 | 0.181 |  | 1.0 |  |  |  | 13.2 | 10 | 0.017 |
|  | chromosome 20 open reading frame 103 (C20irf103) |  | 3.9 | 15 | 0.072 |  | 1.9 | 135 | 0.295 |  | 1.0 |  |  |  | 10.7 | 11 | 0.006 |
| **Locus Coeruleus** | solute carrier family 6 (noradrenalin transporter), member 2 (SLC6A2) | | 171.9 | 1 | 0.004 |  | 103.8 | 3 | 0.010 |  | 145.2 | 1 | 0.006 |  | 1.0 |  |  |
|  | dopamine beta-hydroxylase (DBH) |  | 101.9 | 3 | 0.004 |  | 131.6 | 1 | 0.010 |  | 101.2 | 3 | 0.006 |  | 1.0 |  |  |
|  | transcription factor AP-2 beta (TFAP2B) |  | 13.8 | 6 | 0.008 |  | 17.7 | 5 | 0.010 |  | 19.3 | 6 | 0.006 |  | 1.0 |  |  |
|  | dopa decarboxylase (DDC) |  | 7.5 | 9 | 0.004 |  | 10.4 | 8 | 0.010 |  | 9.3 | 9 | 0.006 |  | 1.0 |  |  |
|  | paried-like(aristaless) homeobox 2a (PHOX2A) |  | 10.3 | 7 | 0.004 |  | 5.3 | 12 | 0.010 |  | 7.2 | 8 | 0.006 |  | 1.0 |  |  |
|  | paried-like homeobox 2b (PHOX2B) |  | 6.3 | 8 | 0.004 |  | 5.6 | 14 | 0.010 |  | 5.9 | 12 | 0.006 |  | 1.0 |  |  |
|  | typrosine hydroxylase (TH) |  | 4.2 | 13 | 0.109 |  | 3.6 | 28 | 0.038 |  | 7.4 | 10 | 0.024 |  | 1.0 |  |  |
|  | alpha-2A- adrenergic ,receptor (ADRA2A) |  | 3.6 | 15 | 0.004 |  | 6.0 | 9 | 0.010 |  | 5.3 | 14 | 0.006 |  | 1.0 |  |  |
|  | solute carrier family 10 (sodium/bile acid cotransporter family), member 4 (SLC10A4) | | 5.0 | 7 | 0.117 |  | 4.9 | 12 | 0.167 |  | 3.0 | 58 | 0.183 |  | 1.0 |  |  |
|  | mab-21-like 2 (MAB21L2) |  | 3.7 | 12 | 0.004 |  | 4.4 | 13 | 0.010 |  | 3.7 | 18 | 0.006 |  | 1.0 |  |  |
|  | prolactin releasing hormone receptor (PRLHR/GPR10) | | 3.7 | 8 | 0.017 |  | 2.9 | 65 | 0.024 |  | 4.1 | 4 | 0.017 |  | 1.0 |  |  |
|  | stonin 1 (STON1/SBLF) |  | 3.0 | 14 | 0.008 |  | 2.6 | 38 | 0.010 |  | 4.5 | 13 | 0.006 |  | 1.0 |  |  |
|  | ionotrophic glutamate receptor, AMPA 4 (GRIA4) |  | 1.9 | 469 | 0.383 |  | 4.7 | 9 | 0.024 |  | 3.4 | 5 | 0.117 |  | 1.0 |  |  |
|  | ankyrin repeat domain 38 (ANKRD38) |  | 1.8 | 198 | 0.017 |  | 4.1 | 7 | 0.024 |  | 3.2 | 3 | 0.017 |  | 1.0 |  |  |

Comparison of microarray expression data across several brain regions in control subjects. The listed genes have enriched expression in the indicated region, as compared to the other regions. For example, PMCH gene expression was increased 73.8 fold in the posterior hypothalamus when compared to the anterior hypothalamus and ranked #1 using the SAM analysis. Similarly, PMCH gene expression in the posterior hypothalamus ranked first versus the diagonal band sample (3.3 fold change), and the LC (275.1 fold change). Only genes enriched in one region versus at least two other regions are listed (see methods).
