# Supplementary material for: IGFBP3 Colocalizes with and Regulates Hypocretin (Orexin)
Source: PLoS One. 2009 Jan 22;4(1):e4254. doi: 10.1371/journal.pone.0004254 (PMC2617764; doi:10.1371/journal.pone.0004254)
Supplement: Table S2 — Overall sleep parameters in mice overexpressing human IGFBP3 (TG) versus wild type littermates (WT)Although total wake is decreased in TG mice, the difference is not statistically significant. Hour by hour analysis revealed that decreased wake occurred prior to light onset (see Fig. 4). (0.06 MB DOC) [file pone.0004254.s002.doc]

**Supplementary Table 2: Overall sleep parameters in mice overexpressing human IGFBP3 (TG) versus wild type littermates (WT)**

|  | WT | TG |
| --- | --- | --- |
| **REM latency (min)** | 6.3 ± 0.6 (9) | 7.0 ± 0.4 (13) |
| **Body weight (g)** |  |  |
| Male | 39.4 ± 1.5 (5) | 35.1 ± 0.7 (5) |
| Female | 33.1 ± 0.8 (7) | 29.9 ± 0.6 (8) |
|  |  |  |
| **Time (%)** |  |  |
| Wake 24h | 54.3 ± 1.8 (9) | 51.3 ± 1.8 (13) |
| NREM 24h | 40.7 ± 1.6 (9) | 43.7 ± 1.7 (13) |
| REM 24h | 5.0 ± 0.4 (9) | 5.1 ± 0.2 (13) |
|  |  |  |
| Wake L | 19.8 ± 1.3 (9) | 18.6 ± 1.0 (13) |
| NREM L | 27.1 ± 0.7 (9) | 27.8 ± 0.9 (13) |
|  |  |  |
| Wake D | 34.4 ± 1.8 (9) | 32.7 ± 1.3 (13) |
| NREM D | 13.6 ± 1.4 (9) | 15.9 ± 1.1 (13) |
|  |  |  |
| **Duration (min)** |  |  |
| Wake 24h | 8.7 ± 1.4 (9) | 8.2 ± 0.9 (13) |
| NREM 24h | 5.2 ± 0.5 (9) | 5.8 ± 0.4 (13) |
| REM 24h | 1.6 ± 0.1 (9) | 1.75 ± 0.04 (13) |
|  |  |  |
| Wake L | 5.0 ± 0.7 (9) | 5.1 ± 0.5 (13) |
| NREM L | 5.4 ± 0.4 (9) | 5.9 ± 0.5 (13) |
|  |  |  |
| Wake D | 16.3 ± 3.5 (9) | 15.9 ± 2.6 (13) |
| NREM D | 5.0 ± 0.7 (9) | 6.0 ± 0.4 (13) |
|  |  |  |
| **Episode counts** |  |  |
| Wake 24h | 105 ± 13 (9) | 97 ± 10 (13) |
| NREM 24h | 121 ± 13 (9) | 114 ± 9 (13) |
| REM 24h | 45 ± 5 (9) | 42 ± 2 (13) |
|  |  |  |
| Wake L | 64 ± 7 (9) | 59 ± 6 (13) |
| NREM L | 76 ± 7 (9) | 71 ± 5 (13) |
|  |  |  |
| Wake D | 41 ± 8 (9) | 38 ± 5 (13) |
| NREM D | 45 ± 8 (9) | 42 ± 5 (13) |

Although total wake is decreased in TG mice, the difference is not statistically significant. Hour by hour analysis revealed that decreased wake occurred prior to light onset (see Fig. 3).
