# Supplementary figures and images for: IGFBP3 Colocalizes with and Regulates Hypocretin (Orexin)
Source: PLoS One. 2009 Jan 22;4(1):e4254. doi: 10.1371/journal.pone.0004254 (PMC2617764; doi:10.1371/journal.pone.0004254)

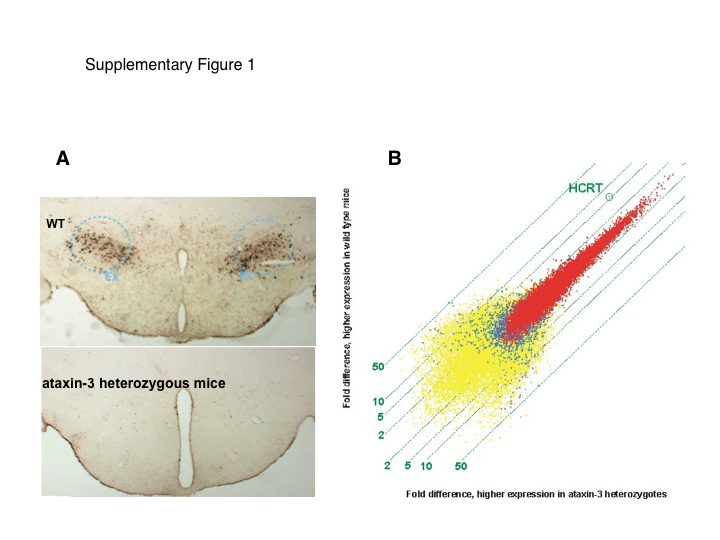

Supplement: Figures S1 — Perifornical hypothalamic region dissected for microarray analysis in mice (A) and transcript abundance distribution plots correlating abundance in wild type versus ataxin-3 transgenic mice (B). (A) Immunocytochemistry of hypocretin in wild type (WT, top) and Hcrt-ataxin-3 transgenic mouse (bottom). The area collected using punches is outlined by a circle in wild type versus transgenic mice lacking most hypocretin cells. Transcript abundance distribution (B) in both genotypes is highly correlated, and hypocretin is one of the outliers (circled dot). For list of differentially regulated transcripts, see Table 3. (1.56 MB TIF) [file pone.0004254.s003.tif]

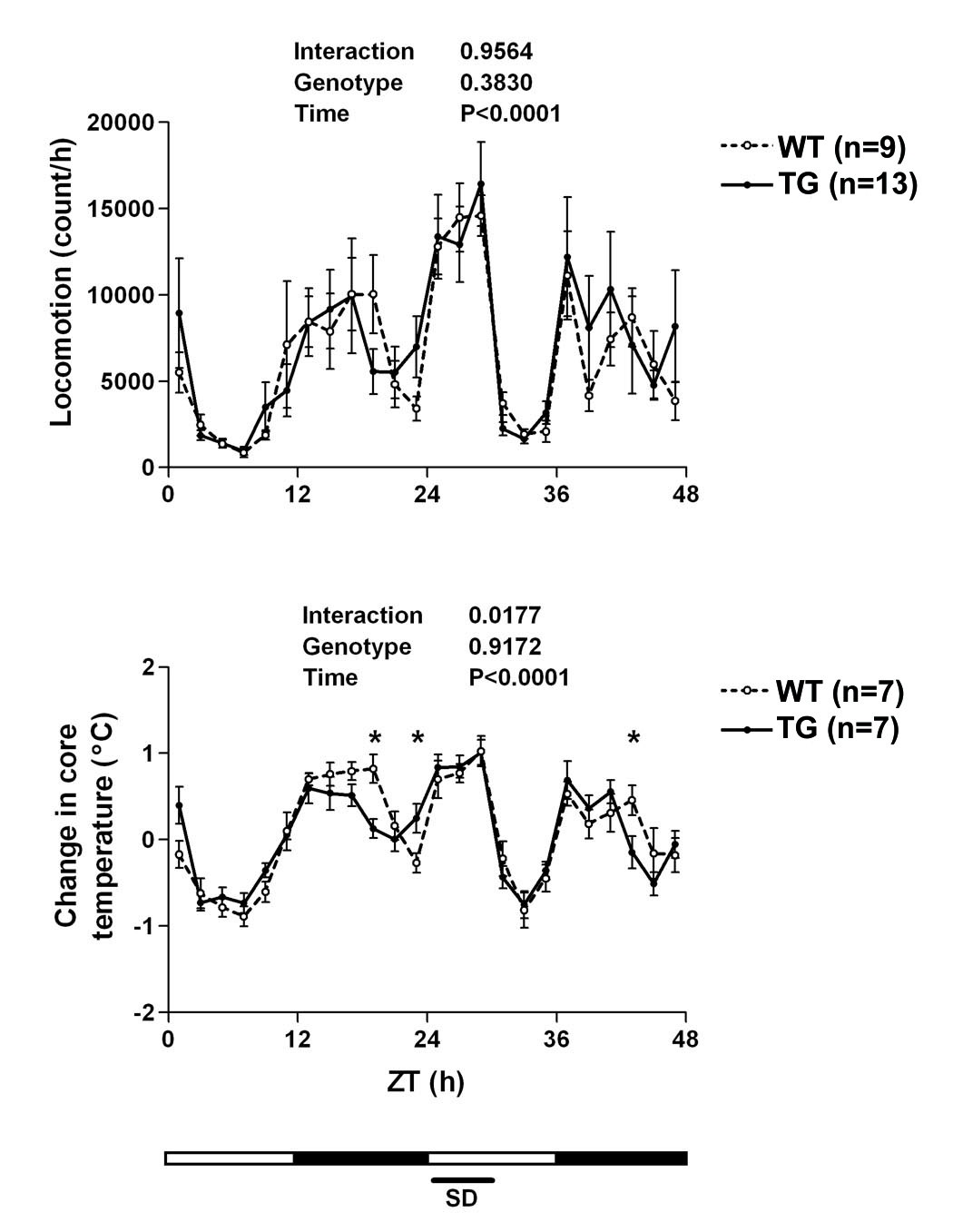

Supplement: Figure S2 — Locomotion (A) and temperature (B) in wild type (WT) versus hIGFBP3 transgenic mice (Tg). Note decreased temperature and locomotion at the end of the active period in hIGFBP3 transgenic mice, mirroring changes in sleep depicted in Fig. 4. (5.70 MB TIF) [file pone.0004254.s004.tif]
